# Contribution of the Ratio of Tocopherol Homologs to the Oxidative Stability of Commercial Vegetable Oils

**DOI:** 10.3390/molecules23010206

**Published:** 2018-01-19

**Authors:** Mathias Zaunschirm, Marc Pignitter, Julia Kienesberger, Natalie Hernler, Christoph Riegger, Manfred Eggersdorfer, Veronika Somoza

**Affiliations:** 1 Department of Physiological Chemistry, Faculty of Chemistry, University of Vienna, 1090 Vienna, Austria; mathias.zaunschirm@univie.ac.at (M.Z.); marc.pignitter@univie.ac.at (M.P.); Julia.Kienesberger@gmx.at (J.K.); hernler.natalie@gmail.com (N.H.); 2 Department of Human Nutrition and Health, DSM Nutritional Products Ltd., 4303 Kaiseraugst, Switzerland; ch.riegger@gmx.ch (C.R.); manfred.eggersdorfer@dsm.com (M.E.)

**Keywords:** vegetable oils, oxidative stability, lipid oxidation, hexanal, tocopherol ratio

## Abstract

The antioxidant activity of tocopherols in vegetable oils was shown to chiefly depend on the amount and the tocopherol homolog present. However, the most effective ratio of tocopherol homologs with regard to the antioxidant capacity has not been elucidated so far. The present study analyzed the effect of different tocopherol concentrations, homologs and ratios of homologs on markers of lipid oxidation in the most commonly consumed vegetable oils (canola, sunflower, soybean oil) stored in a 12 h light/dark cycle at 22 ± 2 °C for 56 days under retail/household conditions. After 56 days of storage, the α-tocopherol-rich canola and sunflower oil showed the strongest rise in lipid peroxides, yielding 25.1 ± 0.03 meq O_2_/kg (+25.3-fold) and 24.7 ± 0.05 meq O_2_/kg (+25.0-fold), respectively. ESR experiments, excluding effects of the oils’ matrices and other minor constituents, confirmed that a food representative tocopherol ratio of (γ + δ)/α = 4.77, as represented in soybean oil, led to a more pronounced delay of lipid oxidation than a lower ratio in canola (1.39) and sunflower oil (0.06). An optimum (γ + δ)/α -tocopherol ratio contributing to the oxidative quality of vegetable oils extending their shelf life has to be investigated.

## 1. Introduction

Tocopherols are characterized by a chromane ring with a hydroxyl group and a hydrophobic side chain. Depending on the number and position of methyl groups on the chromanol ring, tocopherols occur as α-, β-, γ- and δ-homologs, showing structure-specific chemical and biological activities. The hydroxyl group of the chromanol ring chiefly determines the antioxidant activity, as it can donate a hydrogen atom to reduce free radicals. In vivo, the relative antioxidant activity of the tocopherol homologs is generally agreed to follow the order of α > β > γ > δ [1,2,3,4]. In in vitro systems, as well as in foods, the relative antioxidant potency of tocopherol homologs is still under debate, depending on the reaction conditions applied [5,6]. In experiments using triacylglycerol-in-water emulsions or purified canola oil, triacylglycerols demonstrated individual tocopherol homologs to have oxidation protecting properties in the order of δ > γ > α [7,8]. Regarding dietary lipids, in particular fats and oils, either γ- or δ-tocopherol have been reported being the most potent antioxidant [6,7,8,9]. Another aspect that has not been clarified yet is whether not only the quantity of tocopherol homologs but also their ratio to each other determines their antioxidant potential in a food matrix. In addition to the absolute and relative amount of tocopherol homologs, the food matrix, processing and storage conditions, among other factors, have a major impact on the oxidative stability in fats and oils [10,11,12]. For vegetable oils, it is known that up to 32% of the native tocopherols are removed during refining, although, in some cases, re-added after processing [12]. Improper storage, such as exposure to light, oxygen or elevated temperatures, may result in a considerable loss of tocopherols, meaning a decline of bioactive tocopherol homologs, in these products [13], leading to the formation of lipid oxidation products [14]. Whereas the term “loss” describes the decline in bioactive tocopherol homologs during autoxidation of lipids, atmospheric triplet oxygen reacts with lipid alkyl radicals, whose formation is accelerated in the presence of transition metals or heat [15], while photosensitized oxidation of vegetable oils is based on the direct reaction of singlet oxygen (Type II) or superoxide (Type I), released by sensitizers, such as chlorophyll, with unsaturated fatty acids in the presence of light. Both mechanisms, autoxidation as well as photooxidation, result in the formation of primary lipid peroxidation products (lipid hydroperoxides) and, subsequently, to various secondary lipid peroxidation products, such as lipid hydroxides, aldehydes and ketones [16,17]. In one of our previous studies, a substantial loss of α-tocopherol (−58 ± 1.5%), γ-tocopherol (−77 ± 3.5%), and δ-tocopherol (−23 ± 0.7%) was demonstrated in commercial soybean oil after 56 days of retail/household storage (22 °C, 12 h exposure to cold fluorescent light per day) [13]. This decrease in tocopherol homologs was associated with an increase in outcome measures of lipid oxidation, e.g., peroxides and carbon-centered lipid radicals, and a reduced shelf life [13]. The profound loss of tocopherols due to oxidative deterioration under conditions of household storage in soybean oil was unexpected and indicates inaccurate consumer expectations regarding the shelf life of vegetable oils.

Therefore, this study aimed to (i) quantitate tocopherol homologs in canola, corn, olive, soybean and sunflower oil, as commonly consumed dietary oils [18], purchased from local supermarkets in Austria, Bangladesh, Brazil and USA; and to (ii) to explore the impact of tocopherol homologs to the oxidative stability of commercial Austrian oils with a low (sunflower oil), medium (canola oil) and high (soybean oil) (γ + δ)/α -tocopherol homolog ratio stored under private household conditions.

## 2. Results

### 2.1. Quantitation of Tocopherol Homologs and Assessment of the Oxidative Status of Commercial Vegetable Oils

To evaluate the oxidative status of a subset of most commonly consumed commercial vegetable oils [18] at the time of purchase, contents of tocopherol homologs and peroxide values were determined. Quantitation of total tocopherol contents of canola, corn, olive, soybean and sunflower oil revealed a wide range between the five oil types from 331 ± 86.4 mg/kg to 1580 ± 280 mg/kg (Table 1). Moreover, these vegetable oils showed a great variation in their (γ + δ)/α -tocopherol ratio, with canola, corn olive, soybean and sunflower oil exhibiting ratio of 1.55, 1.32, 0.43, 4.29 and 0.20, respectively.

### 2.2. Analysis of the Fatty Acid Profile

The fatty acid composition of the sunflower, canola, and soybean oil was analyzed after purchase (Table 2). Stored oil samples were not subjected to fatty acid analysis since it has been shown recently that the fatty acid composition of soybean oil under comparable storage conditions [13], as well as sunflower oil and canola oil even under accelerated conditions remained unchanged [19,20]. Sunflower oil showed a high amount of oleic acid, which is in accordance with the literature for a high-oleic sunflower oil [21]. Canola [22] and soybean oil [23] also showed a representative fatty acid profile [24] (Table 2).

### 2.3. Quantitation of Tocopherol Homologs in Vegetable Oils Stored under Household Conditions

**Figure 1:**
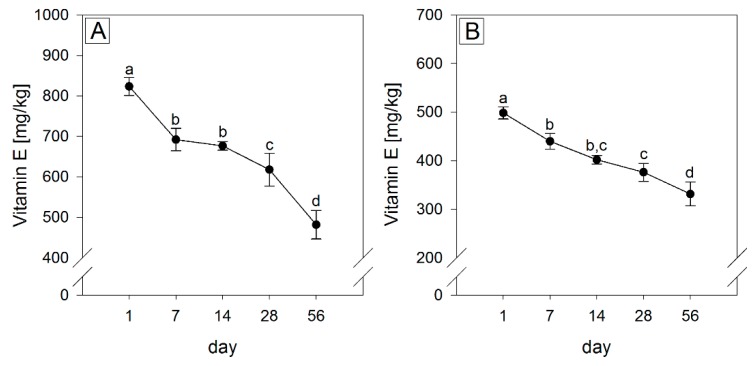

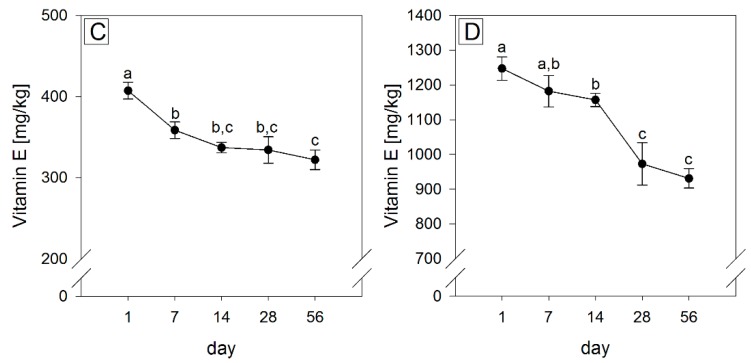
Changes of the total tocopherol contents in different oil types (**A**) sunflower oil; (**B**) canola oil; (**C**) soybean oil and (**D**) fortified soybean oil stored at 22 °C in a 12 h light/dark cycle for 56 days. Data are represented as mean ± SD (*n* = 4). Statistically significant differences were analyzed using one-way ANOVA (*p* ≤ 0.001) followed by Holm-Sidak post hoc test (*p* < 0.05). Different letters indicate significant differences.

Since household storage conditions of soybean oil were demonstrated to decrease contents in tocopherols [13], the stability of tocopherol homologs was assessed in commonly consumed sunflower, canola, soybean oil and fortified soybean oil with a low (0.06), medium (1.39) and high (4.77) (γ + δ)/α -tocopherol ratio, respectively, stored at 22 ± 2 °C alternating 12 h in the light and 12 h in the dark for 56 days (Figure 1). After 56 days of storage, a significant decrease in total tocopherols was seen in sunflower of −42% (from 823 ± 22.0 mg/kg to 481 ± 35.2 mg/kg), canola of −34% (from 498 ± 12.5 mg/kg to 331 ± 24.5 mg/kg), soybean of −21% (from 407 ± 10.4 mg/kg to 322 ± 12.0 mg/kg) and fortified soybean oil of −25% (from 1246 ± 33.6 mg/kg to 931 ± 28.0 mg/kg).

Concentrations of tocopherol homologs also decreased in all oils after 56 days of storage (Table 3), with α-tocopherol being the most affected homolog showing a mean decrease of −42.3 ± 4.16% and −41.0 ± 4.91% in sunflower and canola oil, respectively (*p* < 0.05), whereas the overall mean loss of γ-tocopherol, ranging from −23.8 ± 2.52% to −29.0 ± 2.20%, was less pronounced (*p* < 0.05). Delta-tocopherol, which could be quantitated in soybean oil solely, showed a mean decrease (*p* < 0.05) of 10.9 ± 3.15% and 17.6 ± 2.00% in non- and tocopherol-fortified oil, respectively.

### 2.4. Analysis of POV

**Figure 2:**
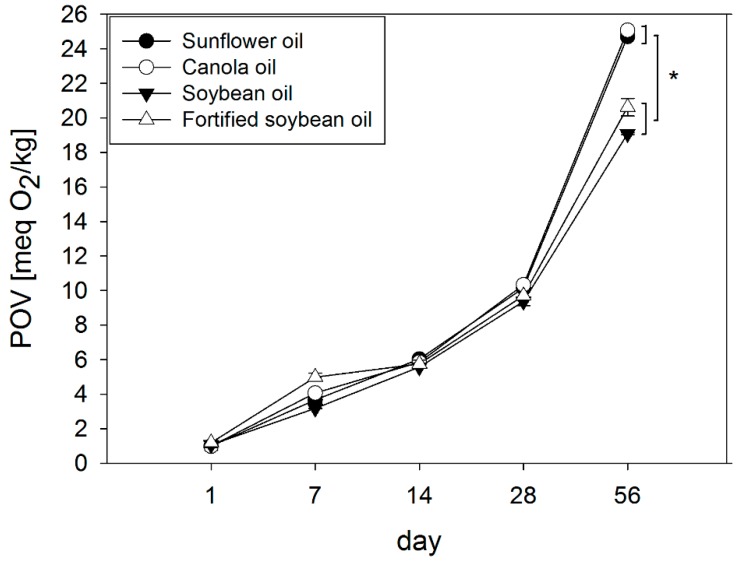
Changes of the peroxide values in four oil types (sunflower, canola, soybean oil, fortified soybean oil) stored at 22 °C in a 12 h light/dark cycle for 56 days. Data are represented as mean ± SD (*n* = 4). Statistically significant differences between the different oil types on day 56 were analyzed using one-way ANOVA (*p* ≤ 0.001) followed by Holm-Sidak post hoc test (* *p* < 0.05).

After 56 days of storage, the POV increased in sunflower oil to 24.7 ± 0.05 meq O_2_/kg (+2395%), in canola oil to 25.1 ± 0.03 meq O_2_/kg (+2425%), in soybean oil to 19.1 ± 0.05 meq O_2_/kg (+1659%) and fortified soybean oil to 20.6 ± 0.51 meq O_2_/kg (+1631%) compared to day 1 (*p* < 0.05) (Figure 2). Sunflower and canola oil, both high in α-tocopherol with 235 mg/kg and 788 mg/kg respectively, didn’t differ from soybean and fortified soybean oil in their initial peroxide values, but showed about 35% and 20% higher POVs compared to soybean and fortified soybean oil after 56 days of storage, respectively (*p* < 0.05).

### 2.5. Correlation Analyses between POVs and Tocopherols

Pearson correlation analyses of total tocopherols, tocopherol homologs and POVs on the last day of the storage period revealed a significant association between an increase in the POV and the decrease in total tocopherols as well as α-tocopherol after 56 days of storage (Table 4). The highest correlation coefficient was seen between the increase in the POV and the tocopherol ratio of (γ + δ)/α.

### 2.6. Analyses of Free Radical-Scavenging Activity of α- γ- and δ-Tocopherol

**Figure 3:**
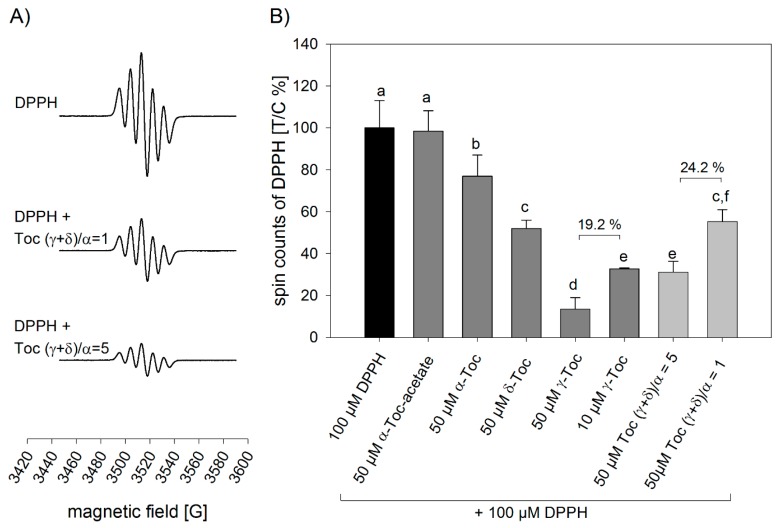
(**A**) ESR signals of DPPH and DPPH radical scavenging activity of 50 µM tocopherol ratio (γ + δ)/α = 1 and 50 µM tocopherol ratio (γ + δ)/α = 5 and (**B**) spin counts of DPPH. After three minutes spin counts of 100 µM DPPH (control = 100%) alone or in presence of 50 µM α-tocopheryl acetate (α-Toc acetate), 50 µM α-tocopherol (α-Toc), 50 µM δ-tocopherol (δ-Toc), 50 µM γ-tocopherol (γ-Toc), 10 µM γ-tocopherol (γ-Toc), 50 µM tocopherol ratio (γ + δ)/α = 5 and 50 µM tocopherol ratio (γ + δ)/α = 1 were analyzed. Data are presented as mean ± SD (*n* = 3). Statistically significant differences were analyzed using one-way ANOVA (*p* ≤ 0.001) followed by Holm-Sidak post hoc test (*p* < 0.05). Different letters indicate significant differences.

To confirm the antioxidant properties of the tocopherol homolog ratio (γ + δ)/α and to exclude possible effects of the oils’ matrices including potential effects of minor constituents, the free radical scavenging activity of individual tocopherol homologs and combinations thereof was investigated by means of electron spin resonance (ESR) spectroscopy (Figure 3). At the concentrations of 50 µM and 10 µM, γ-tocopherol showed the strongest scavenging activity towards DPPH radicals with −87% and −67%, followed by 50 µM δ-tocopherol (−48%) and 50 µM α-tocopherol (−23%). Alpha-tocopheryl acetate, as reported [25], did not reduce the spin counts of the DPPH radical. A mixture of α-, γ- and δ-tocopherol in a ratio of (γ + δ)/α = 5 showed a higher scavenging activity (−69%) than the tocopherol homolog ratio (γ + δ)/α = 1 (−45%) (*p* < 0.05), but there was no statistically significant difference to that of 10 µM γ-tocopherol. Furthermore, it was shown that tocopherols in a tocopherol homolog ratio (γ + δ)/α = 1 did not show a significant difference in scavenging the DPPH radicals compared to δ-tocopherol when applied in concentrations of 50 µM. The highest scavenging activities were demonstrated for 50 µM of γ-tocopherol (−86.5%) (*p* < 0.05). Examining whether the tocopherol homolog ratio of (γ + δ)/α = 5 showed a stronger effect in scavenging the DPPH radicals than only γ-tocopherol, the scavenging activities of γ-tocopherol were also compared in the ratio of 5:1. However, the tocopherol homolog ratio of (γ + δ)/α = 5 showed a decrease of the spin counts of the DPPH radical of 24.2% compared to tocopherol homolog ratio (γ + δ)/α = 1, whereas 50 µM γ-tocopherol showed only a 19.2% decrease compared to 10 µM γ-tocopherol.

### 2.7. Quantitation of Hexanal

**Figure 4:**
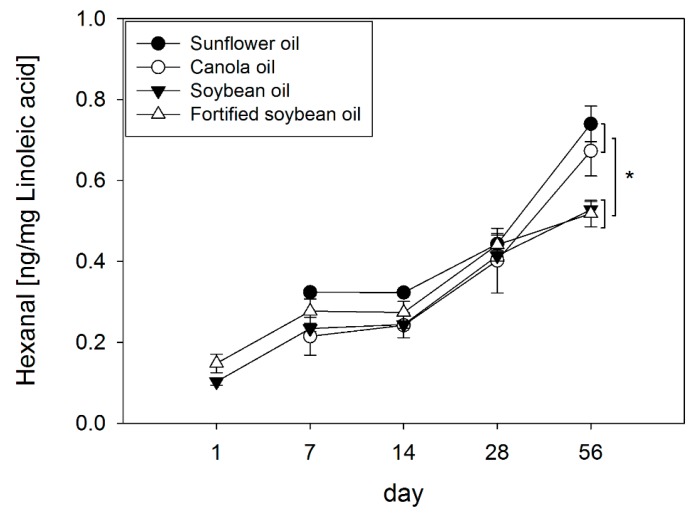
Changes of the hexanal content normalized to the linoleic acid concentration of each oil type (sunflower, canola, soybean oil, fortified soybean oil) after storage at 22 °C in a 12 h light/dark cycle for 56 days. Data are represented as mean ± SD (*n* = 3–4). Statistically significant differences between the different oil types on day 56 were analyzed using one-way ANOVA (*p* ≤ 0.001) followed by Holm-Sidak post hoc test (* *p* < 0.05). Hexanal concentrations in sunflower and canola oil were below the limit of quantification on day 1.

Hexanal, mainly formed by decomposition of linoleic acid hydroperoxides, was analyzed as a secondary lipid peroxidation marker [26]. After normalizing the hexanal content of each oil to its linoleic acid (LA) concentration, results showed increasing hexanal concentrations in sunflower oil from 0.32 ± 0.01 ng/mg LA to 0.74 ± 0.04 ng/mg LA, in canola from 0.22 ± 0.05 ng/mg LA to 0.67 ± 0.06 ng/mg LA, in soybean oil from 0.10 ± 0.01 ng/mg LA to 0.53 ± 0.02 ng/mg LA and in fortified soybean oil from 0.15 ± 0.02 ng/mg LA to 0.52 ± 0.03 ng/mg LA after storage of 56 days (Figure 4).

Over the storage period accelerated contents of hexanal [ng/mg LA] could be shown in all four oil types compared to their initial concentrations (*p* < 0.05). The formation of hexanal [ng/mg LA] was shown to be statistically significant higher in sunflower (+140% and +142%) and canola oil (+126% and +129%) compared to soybean and fortified soybean oil showing the highest hexanal concentrations in the following order sunflower = canola > soybean = fortified soybean oil at the end of the experimental period (*p* < 0.05).

## 3. Discussion

Vegetable oils are prone to oxidative deterioration, which is determined by the oils’ fatty acid composition and its content in antioxidants [27], of which tocopherols play a pivotal role [10]. The contribution of tocopherols to the oxidative stability of vegetable oils has recently been studied by analyzing the ESR spectrum of galvinoxyl radicals in the presence of commercial olive, canola, soybean, sunflower, sesame and walnut oils purchased from local supermarkets “off-shelf” [28]. Concentrations of α-tocopherol and γ-tocopherol homologs correlated well with the reduction of galvinoxyl radicals and contributed between 20% (olive) and 85% (soybean) to the antioxidant potential, depending on type of oil. However, a correlation between galvinoxyl radical reduction and the concentration of total phenolic compounds was not demonstrated, pointing to the role of tocopherols as antioxidants.

However, despite comprehensive research in this field, the contribution of individual tocopherol analogs to the oxidative stability of vegetable oils stored under household conditions has not been fully elucidated yet. Among the tocopherols, β-tocopherol was not analyzed because of its low abundance in the oil types tested [12,29,30]. Previous studies have shown that the concentrations of β-tocopherol in the tested oil types were below the limit of detection (0.2 ± 0.02 µg/mL) or the limit of quantification (0.67 ± 0.07 µg/mL) [29,31,32]. Moreover, it has been shown that β-tocopherol in very low concentrations could only be detected in soybean oil on day 1 during a storage period of 56 days, indicating a relatively rapid degradation of this homolog [31]. Analyses of the purchased products from retail stores in Austria, Bangladesh, Brazil and the USA revealed an already well-known spectrum of fatty acids [13,21,33] and a wide concentration range of total tocopherol and tocopherol homologs [32,34,35,36], which is likely due to different cultivars or growing areas, differences in the manufacturing process and/or tocopherol fortification after refining [37,38]. High concentrations of α-tocopherol, being the most nutritionally active tocopherol homolog with respect to the vitamin E activity [1], were quantitated in sunflower and corn oil, whereas soybean oil contained high amounts of γ- and δ-tocopherol. At the time of purchase, oil samples were analyzed for POVs below 10 meq O_2_/kg oil, which was expected as oils were purchased about 7.5 months prior to their expiry date [39]. After quantitation of tocopherols and POVs in the commercial “off-shelf” samples from Austria, Bangladesh, Brazil and the USA (Table 1), the contribution of individual tocopherol homologs to the oxidative stability was analyzed during conditions of household storage of 56 days (exposure to cold fluorescent light for 12 h a day and intermittent openings). Oil samples (sunflower oil, canola oil, soybean oil and fortified soybean oil) were selected (Table 1) since these oil types represent a wide range of tocopherol homolog ratios and are the most widely consumed vegetable oils worldwide that reach the consumer as minimally-processed oils, whereas palm oil, on the other hand, also produced in high amounts worldwide, is mainly consumed with processed foods. Moreover, canola and sunflower oil are the most consumed vegetable oils in Europe [18]. All samples were purchased from local Austrian supermarkets and had 7.5 ± 0.5 months left to reach the end of their expiry dates. Their initial tocopherol contents were within the range found in the literature (Figure 1) [32,34,35,36], although the α-tocopherol concentration in the sunflower oil was at the low end of this range. A substantial loss of total tocopherols, with the greatest decrease in sunflower oil (−42%), was detected. These results are in accordance with previous studies, which could also show a substantial loss of tocopherols in sunflower [40], canola [41] and soybean oil [13] after storage at ambient temperature in the light.

In this study, it was shown that after 56 days of storage under light/dark conditions at 22 °C with intermittent openings, the loss of total tocopherols in the tested oils followed the order of sunflower oil > canola oil > soybean oil = fortified soybean oil (*p* < 0.05). Since it has been shown that the fatty acid composition in sunflower (14 days, 80 °C) and canola (15 days, 60 °C) oil did not change under accelerated storage conditions [19,20,42,43] and, in soybean oil, under comparable (56 days, 22 °C) or accelerated (56 days, 32 °C) conditions [13], quantitative changes to the fatty acid composition are not a sensitive outcome measure for determining the oxidative status of the tested vegetable oils stored under household conditions. Instead, POV analyses revealed a corresponding increase in all four oils, with sunflower and canola oil, both rich in α-tocopherol with 235 ppm and 788 ppm respectively, showing POVs which were 35% and 20% higher than those analyzed for soybean and fortified soybean oil, respectively. This could be explained by the initially higher amount of unsaturated fatty acids in sunflower and canola oil. Remarkably, all oils showed POVs higher than 10 meq O_2_/kg oil after 28 days of storage, which is the maximum concentration of POVs recommended for edible oils by the Codex Alimentarius [39]. With respect to the tocopherol homologs, our findings indicate a lower antioxidant potential of α-tocopherol compared to γ- and δ-tocopherol in vegetable oils, which has been reported earlier [7,8]. It has also been shown that α-, γ- and δ-tocopherol homologs showed different antioxidant properties, depending on their concentrations in soybean oil stored above room temperature, e.g., 40–60 °C [9,44]. Back in 1994, Huang et al. [45] demonstrated the formation of hydroperoxides in bulk oil to increase with rising α-tocopherol concentrations from 250 to 1000 ppm, whereas γ-tocopherol showed an antioxidant potential in that concentration range.

In this study, vegetable oils with distinct tocopherol homolog contents showed a different decrease in α-, γ- and δ-tocopherols over the storage period of 56 days in the transparent PET bottles, exposed to cold fluorescent light for 12 h daily and intermittently opened to increase the oxygen content in the headspace, as practiced during household handling. The most pronounced loss of α-tocopherol in sunflower and canola oil might be explained by their high contents in α-tocopherol. Alpha-tocopherol is known to form α-tocopheroxyl radicals and, as such, react with lipid peroxyl radicals, resulting in the formation of α-tocopherol (epoxy) quinones and alkoxyl radicals, which are known to initiate lipid peroxidation. Otherwise, α-tocopheroxyl radicals may react with either unsaturated fatty acids or lipid hydroperoxides, thereby leading to the formation of lipid radicals or lipid peroxyl radicals [5]. Gamma- and δ-tocopherol also scavenge lipid peroxyl radicals, generating γ- and δ-tocopheroxyl radicals, which could further react with another tocopherol molecule to form dimers or trimers, or react with lipid peroxyl radicals to tocopherol quinones [46], thereby terminating lipid oxidation. The here presented results support the higher antioxidant potential of γ- and δ-tocopherol compared to α-tocopherol to delay the increase in POVs in vegetable oils, as shown by Isnardy et al. [7]. Correlation analyses between changes in POVs and individual tocopherol homologs revealed a significant correlation. The highest correlation coefficient (r = −0.97, *p* < 0.001) was achieved for a correlation between the increase of POV and a (γ + δ)/α -tocopherol ratio. The sunflower and canola oil samples, both rich in α-tocopherol, showed a very low (γ + δ)/α -tocopherol ratio of 0.06 and 1.39, respectively, compared to soybean oil and the fortified soybean oil, which had a (γ + δ)/α -tocopherol ratio of 4.77 and 4.90, respectively. Moreover, correlation analyses of the initial P/S ratio and the (γ + δ)/α -tocopherol ratio of sunflower, canola and soybean oil showed a strong positive correlation (r = 0.92, *p* < 0.001), indicating that the soybean oil naturally might show a better (γ + δ)/α -tocopherol ratio than sunflower and canola oil.

ESR experiments, eliminating possible effects of the oils’ matrices and other minor components, supported this hypothesis, showing that a tocopherol ratio (γ + δ)/α of 5 showed a significantly higher DPPH-radical scavenging activity than a tocopherol ratio (γ + δ)/α of 1. The radical scavenging effect of the tocopherol ratio (γ + δ)/α of 5 was similar to that of 10 µM γ-tocopherol, which has been demonstrated to have a higher antioxidant potency in vegetable oils than α-and δ-tocopherol [47]. The decreased scavenging activity of α-tocopherol may be explained by the number and position of the methyl groups in the homolog leading to a positive inductive effect, which has been shown previously [48]. Here, it could be shown that a tocopherol ratio (γ + δ)/α = 5 reduced the spin counts of a DPPH radical by 24.2% compared to a tocopherol ratio of (γ + δ)/α = 1, and that 50 µM γ-tocopherol led to a reduction of 19.2% compared to 10 µM γ-tocopherol, pointing out the importance of γ- and δ-tocopherols in the (γ + δ)/α -tocopherol ratio. This supports our findings that the ratio of (γ + δ)/α -tocopherol plays an important role in the progression of lipid oxidation. Although soybean oil contains highest amounts of linoleic acid among the study oils, it shows the most favorable tocopherol ratio of all oils, thereby explaining its higher oxidative stability. Confirming the postulated antioxidant potency of a higher food representative (γ + δ)/α -tocopherol ratio, it was shown that hexanal concentrations, after long term storage of 56 days, were significantly higher in sunflower and canola oil, showing a lower (γ + δ)/α -tocopherol ratio as compared to soybean and fortified soybean oil. Moreover, correlation analysis showed a negative relationship of the (γ + δ)/α -tocopherol ratio and the relative formation of hexanal after the storage period (r = −0.94, *p* < 0.001), supporting our findings that a higher food representative (γ + δ)/α -tocopherol ratio could contribute to the oils’ oxidative stability. This shows that not only the amount of formation of primary but also of secondary lipid peroxidation products is affected by the (γ + δ)/α -tocopherol ratio.

For vegetable oils, which always contain a mixture of naturally occurring tocopherol homologs, the here proposed (γ + δ)/α -tocopherol ratio might help to better estimate and optimize their oxidative stability during household storage. A possible approach to enhance the contents of γ- and δ-tocopherol in oil seeds could be a modulation of the expression of key enzymes involved in α-tocopherol synthesis, such as tocopherol cyclase or γ-tocopherol methyltransferase, by selective breeding of mutated varieties or metabolic engineering [49,50,51,52]. Furthermore, blending vegetable oils, having a low (γ + δ)/α -tocopherol ratio, with vegetable oils, high in γ- and δ- tocopherols, could be a favorable way to improve the shelf life of vegetable oils during household handling. In any case, α-tocopherol should not be removed since this tocopherol homolog presents the highest Vitamin E activity. Since only one sample per oil type was tested in this mechanistic study, the results may not be generalizable. This study primarily focused on the impact of tocopherol homologs on the oxidative stability of vegetable oils. The role of other oil constituents on the oxidative stability has to be addressed in future studies.

From a nutritional point of view, α-tocopherol is the most valuable tocopherol homolog since it shows the highest vitamin E activity. Given the limited oxidative stability of α-tocopherol, α-tocopheryl acetate is widely used in food fortification programs [53], whereas it has no protecting effect against photo- and autoxidation [25]. That said, strategies to increase the oxidative stability of vegetable oils containing high amounts of α-tocopherol are needed to guarantee appropriate vitamin E contents in vegetable oils as major dietary sources of this vitamin.

## 4. Materials and Methods 

### 4.1. Chemicals 

dl-α-tocopherol, dl-α-tocopheryl acetate, (+)-γ-tocopherol and (+)-δ-tocopherol were bought from Sigma Aldrich (Vienna, Austria). Hexanal-d_12_ (98.5 atom% D) was purchased from C/D/N Isotopes Inc. (Pointe-Claire, QC, Canada) and all other chemicals were ordered from Sigma-Aldrich (Vienna, Austria) or Carl Roth (Karlsruhe, Germany).

### 4.2. Oil Samples and Study Design 

**Figure 5:**
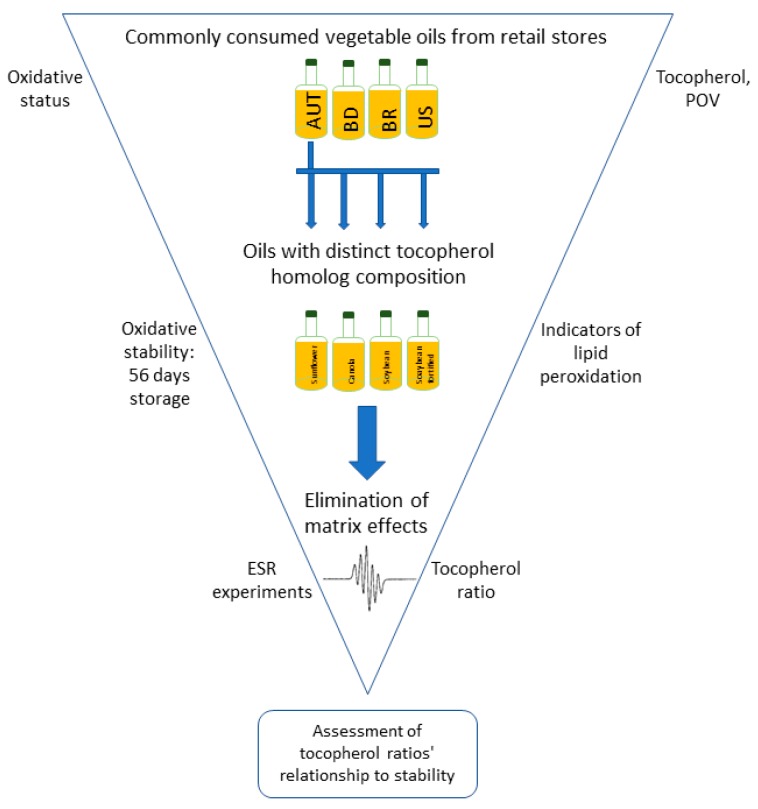
Study design.

From retail stores in Austria (AT), Bangladesh (BD), Brazil (BR) and the United States (US), a total of 55 vegetable oils of five oil types (olive [AT: *n* = 18], canola [AT: *n* = 11, US: *n* = 4], corn [AT: *n* = 2], sunflower [AT: *n* = 5], soybean [BD: *n* = 3, BR: *n* = 8, US: *n* = 4]) was purchased. Tocopherol contents and peroxide values (POVs) of the oil samples were analyzed. After quantitation of the tocopherol homologs in the market samples, we chose a refined sunflower oil (AT, rich in α-tocopherol: 788 ± 20.6 mg/kg oil), a refined canola oil (AT, showing similar amounts of α- and γ-tocopherol: 235 ± 6.16 mg/kg oil and 263 ± 6.44 mg/kg oil, respectively), and a refined soybean oil (AT, with dominating concentrations of 273 ± 6.76 mg/kg oil γ-tocopherol over α- (68.9 ± 2.97 mg/kg oil) and δ-tocopherol (64.8 ± 0.87 mg/kg oil) for a storage experiment under typical household conditions [13]. The soybean oil, as one of the most consumed vegetable oils worldwide [18], was supplemented with a 3-fold addition of α-, γ- and δ-tocopherol concentrations to elucidate the impact of increased concentrations of individual tocopherols when their ratio remains constant. A total of 460 ± 0.1 g of each oil sample was transferred into transparent 0.5 L PET (polyethylene terephthalate) bottles (Radlberger, Unterradlberg, Austria) and stored closed at 22 ± 2 °C, alternating 12 h in the dark and 12 h in the light for 56 days. Analyses of the oil samples were performed on day 1, 7, 14, 28 and 56 by withdrawing 40 mL oil from each bottle every analysis day. In order to mimic household conditions, a total volume of 40 mL of each oil sample was quoted on day 35, 42 and 59. Lamps emitting cold fluorescent light were used as the light source (Tornado cool daylight, 1450 lm, 103 W, UV spectrum: 400–650 nm, Philips, Eindhoven, The Netherlands) [13]. The lamps were fixed 34 cm above the bottles, which were placed in 15 cm distance to each other. All oil samples collected were stored at −80 °C until analyses. The fatty acid profile was analyzed and, as outcome measures of lipid oxidation, the POVs, hexanal concentrations and the contents of tocopherol homologs were analyzed on day 1, 7, 14, 28 and 56. All commercial oils were purchased and analyzed within their expiry dates, and had 7.5 ± 0.5 months left to reach the end of their expiry date at the end of the study protocol (56 days of storage) (Figure 5).

### 4.3. Quantitation of Fatty Acid Composition

The fatty acid composition of the different oil types was analyzed using a GC-FID as reported earlier [13]. Briefly, to 100 mg oil sample, 500 µL heptadecanoic acid methyl ester (1%) was added as an internal standard. After dissolving in 2 mL toluene, 100 mg pyrogallol was added as an antioxidant. For methylation of the fatty acids, 4 mL of a 0.5 M sodium methoxide solution was added to the mixture, purged with nitrogen for 2 min and kept in a water bath at 50 °C for 10 min. After cooling down the solution at 4 °C for 5 min, 200 µL glacial acetic acid, 5 mL double-distilled water and 5 mL hexane were added, vortexed for 2 min and extraction was performed. Instrument parameters of the GC-FID (Shimadzu GC-2010 Plus, Vienna, Austria) for analysis of the fatty acid methyl esters (FAMEs) and further quantitation were chosen according to Pignitter and colleagues [13]. The fatty acid methyl esters of the different oil types were quantitated with internal standard using representative FAMEs for calibration.

### 4.4. Quantitation of Tocopherols

Tocopherols were analyzed according to Pignitter et al. [31]. Briefly, 0.05 g of oil sample was dissolved in 1 mL 2-propanol, vortexed and filtered through a nylon filter (0.22 µm). Analysis was carried out by means of a UHPLC (Dionex Ultimate 3000 LC system, Thermo Fisher Scientific, Vienna, Austria) with a flow rate of 1 mL/min using a C18 column (Kinetex EVO, 150 × 4.6 mm, 5 µm, Phenomenex). For separation of the tocopherols, a methanol gradient was used, starting with 75% methanol and 25% double-distilled water, reaching 100% methanol after 10 min and holding the plateau for 3 min. The initial conditions were reached again at 15 min. The tocopherol homologs were detected at a wavelength of 295 nm and quantitated using the standard addition method. Total tocopherol content was calculated by summing up the concentrations of α-, γ- and δ-tocopherol homolog. Limit of detection of α-, γ- and δ-tocopherol was determined by a signal-to-noise ratio of 3 with 0.30 µg/mL, 0.53 µg/mL and 0.23 µg/mL, respectively.

### 4.5. Analysis of the Peroxide Value

Determination of the peroxide value (POV) was performed according to the AOCS official method Cd 8-53 [54]. Briefly, after dissolving 5.0 g oil sample in 30 mL of a mixture of glacial acetic acid and chloroform (3:2, *v*/*v*), 0.5 mL of a saturated potassium iodide solution was added and mixed well for 1 min. Afterwards, 30 mL of double-distilled water was added and an end point titration with 0.1 N sodium thiosulfate, with starch as an indicator, was performed.

### 4.6. Measurement of Free Radical-Scavenging Activity

The free radical-scavenging activity of the tocopherol homologs was evaluated by ESR (electron spin resonance) spectroscopy. The stable free radical 2,2-diphenyl-1-picrylhydrazyl (DPPH) at a concentration of 100 µM was monitored in the presence or absence of 50 µM α-tocopheryl acetate, 50 µM α-tocopherol, 50 µM γ-tocopherol, 50 µM δ-tocopherol, 10 µM γ-tocopherol, 50 µM tocopherol ratio (γ + δ/α) = 1 and 50 µM tocopherol ratio (γ + δ/α) = 5 in the dark. DPPH, tocopherol and α-tocopheryl acetate were dissolved in ethanol/water (1:1, *v*/*v*), and transferred to a capillary tube (100 µL), which was placed into a high sensitivity resonator (Bruker ER 4122SHQE). The stability of DPPH was examined after 3 min by a cw-ESR spectrometer (Elexsys-II E500, Bruker Biospin, Rheinstetten, Germany) operating at 9 GHz with 100 kHz modulation frequency at 25 °C. DPPH spectra were recorded by setting the following parameters: center field, 3518.35 G; sweep field, 144 G; sweep time, 300 s; conversion time, 292.97 ms; modulation amplitude, 4 G; microwave power, 4 mW; receiver gain, 99 dB; resolution, 1024 points; scan time, 5 min. The two neighboring nitrogen atoms resulted in five hyperfine splitting lines with isotropic hyperfine coupling constants of approximately 8.2 and 9.3 G. For quantitative measurements of the spin counts of DPPH (Ns), the DPPH spectrum was double-integrated (DI). A reference-free quantitation of the number of spins was performed according to the following equation considering the sample volume (V), the microwave power (P), the modulation amplitude (Bm), the Q-factor (Q) of the resonator, the calibration factor (c) of the resonator, the electron spin (S), the Boltzmann factor (n_B_) and the resonator field profile (f (B_1_, Bm)) [55]:

Ns = DI × V × [P_1/2_ × B_m_ × Q × c × S × (S + 1) × n_B_ × f (B_1_, Bm)]^−1^

### 4.7. Quantitation of Hexanal

Hexanal was quantitated as reported previously [31]. Briefly, in a 20-mL brown glass vial, 2.5 g of oil sample was dissolved 1:1 in methanol/double-distilled water (3:1, *v*/*v*) and 0.4 µg/mL hexanal-d_12_ was added as an internal standard. After sealing airtight the vial, the sample was incubated on a CTC Combi Pal (CTC Analytics AG, Zwingen, Switzerland) and analysis was performed by means of a GC-MS (GCMS-QP 2010 Ultra, Shimadzu, Vienna, Austria) using a capillary column (ZB-WAX Zebron, 30 m × 0.25 mm i.d., 0.25 μm film thickness) for separation. Detailed measurement parameters are given by Pignitter et al. [31]. Quantitation of hexanal was carried out using stable isotope dilution analysis selecting fragment ions *m*/*z* 72 for hexanal and *m*/*z* 80 for hexanal-d_12_ in SIM mode. Hexanal and hexanal-d_12_ in the sample were identified by comparing their retention times and fragmentation patterns with their corresponding reference standards. In addition, hexanal was identified using the NIST11 database by comparing its fragmentation pattern with a reference spectrum. Limit of detection was determined by a signal-to-noise ratio of 3 with 0.01 µg/mL.

### 4.8. Statistical Analysis

Statistical analyses were carried out using SigmaPlot 11 (Systat Software Inc., Chicago, IL, USA). One-way ANOVA followed by Holm-Sidak post hoc test was used for determining statistical difference. Correlation analyses were done using the Pearson calculation method. Statistical analyses were carried out with three to four independent replicates per oil type (*n* = 3–4) and two technical replicates (for POV analysis one technical replicate). Outliers were eliminated using the Nalimov outlier test. *p* values below 0.05 indicate statistical significance. Data are displayed as mean ± standard deviation (SD).

## 5. Conclusions

The here-presented results as well as recent findings of our group in soybean oil [13] clearly demonstrate that household handling of vegetable oils results in a severe loss of tocopherols and in a considerable rise of peroxides, reducing the oil’s shelf life. After 28 days of storage, all oils demonstrated POVs higher than 10 meq O_2_/kg oil, which is the maximum concentration recommended by the Codex Alimentarius for edible oils. As a consequence, the guidelines for defining the expiry dates of vegetable oils subjected to household use should be re-evaluated. From a nutritional perspective, a vegetable oil should provide high amounts of α-tocopherol as the tocopherol homolog with the highest vitamin E activity. However, concentrations of γ- and δ-tocopherols should be adjusted in a distinct ratio of (γ + δ/α) -tocopherol to optimize the oxidative stability of the oil during household storage conditions in order to guarantee not only sensory properties but also the nutritional quality of the product. Measures to improve the oxidative stability of vegetable oils by optimizing the tocopherol homolog composition towards an ideal (γ + δ/α) -tocopherol ratio should be implemented in plant production and processing, although the contribution of other intrinsic factors, e.g., fatty acid composition, other antioxidants, mineral contents or pro-oxidants, on the oxidative stability of vegetable oils must still be elucidated in future studies.

## Figures and Tables

**Table 1:** Quantitation of tocopherols and POV of five commercial oil types obtained local markets in Bangladesh, Brazil, Austria and the USA ^a^.

	Canola Oil (*n* = 15)	Corn Oil (*n* = 2)	Olive Oil (*n* = 18)	Soybean Oil (*n* = 15)	Sunflower Oil (*n* = 5)
Tocopherols					
(mg/kg)					
α	312 ± 70.2	682 ± 143	290 ± 37.2	215 ± 34.9	648 ± 127
γ	389 ± 72.7	793 ± 126	126 ± 11.2	659 ± 121	131 ± 2.50
δ	95.2 ± 5.40	104 ± 10.8	n.d.	264 ± 35.6	n.d.
total	714 ± 139	1580 ± 280	331 ± 86.4	1138 ± 167	713 ± 160
POV					
(meq O_2_/kg)	2.49 ± 1.03	2.35 ± 0.8	6.70 ± 2.34	1.87 ± 0.64	2.66 ± 0.98

^a^ Results are displayed as mean ± SD. n.d. = not detectable.

**Table 2:** Fatty acid profile of the tested vegetable oils ^d^.

Fatty Acid	Sunflower Oil	Canola Oil	Soybean Oil
		[Relative %]	
C14:0	n.d.	0.07 ± 0.01	n.d.
C16:0	4.47 ± 0.16 ^a^	4.89 ± 0.72 ^b^	11.5 ± 0.14 ^c^
C18:0	2.82 ± 0.15 ^a^	1.72 ± 0.28 ^b^	3.62 ± 0.14 ^a^
C18:1	76.7 ± 3.47 ^a^	63.9 ± 10.3 ^a^	24.0 ± 0.47 ^c^
C18:2	15.4 ± 0.61 ^a^	20.5 ± 3.24 ^a^	54.6 ± 0.63 ^b^
C18:3	0.20 ± 0.01 ^a^	6.37 ± 0.71 ^b^	5.87 ± 0.07 ^b^
C20:0	0.34 ± 0.01 ^a^	0.60 ± 0.10 ^b^	0.46 ± 0.03 ^a,b^
C22:0	n.d.	1.04 ± 0.07	n.d.
C22:1	n.d.	0.71 ± 0.03	n.d.
C24:0	n.d.	0.17 ± 0.04	n.d.
P/S ratio	2.04 ^a^	3.16 ^b^	3.88 ^c^

^d^ Data are displayed as mean ± SD (*n* = 3–4). n.d. = not detectable. Statistically significant differences were analyzed using one-way ANOVA (*p* ≤ 0.001), followed by Holm-Sidak post hoc test (*p* < 0.05). Different letters indicate significant differences in the relative amount of a fatty acid or the P/S ratio between the oil types.

**Table 3:** Changes of tocopherol homologs in different oil types stored in a 12 h light/dark cycle at 22 ± 2 °C for 56 days ^e^.

Oil Type	Tocopherols	Day 1	Day 7	Day 14	Day 28	Day 56	Day 1–56
	(mg/kg)						−Δ [%]
Sunflower	α	788 ± 20.6	660 ± 29.2 *	642 ± 11.7 *	587 ± 40.3 *	455 ± 32.8 *	42.3 ± 4.16 ^a^
	γ	35.7 ± 2.63	31.3 ± 1.65	34.0 ± 1.55	30.2 ± 0.74 *	26.9 ± 2.85 *	24.7 ± 8.00 ^b,d^
Canola	α	235 ± 6.16	198 ± 13.9 *	181 ± 4.28 *	170 ± 7.71 *	138 ± 11.5 *	41.0 ± 4.91 ^a^
	γ	263 ± 6.44	242 ± 2.82	221 ± 4.64 *	206 ± 10.8 *	193 ± 13.0 *	26.8 ± 4.95 ^b,d^
Soybean	α	68.9 ± 2.97	59.7 ± 1.71 *	57.9 ±1.74 *	57.6 ±3.36 *	55.9 ± 3.29 *	19.0 ± 4.78 ^b,c,d^
	γ	273 ± 6.76	239 ± 6.05 *	221 ± 4.68 *	217 ± 10.5 *	208 ± 6.89 *	23.8 ± 2.52 ^b,d^
	δ	64.8 ± 0.87	60.1 ± 2.66 *	58.2 ± 0.68 *	59.4 ± 2.47 *	57.8 ± 2.04 *	10.9 ± 3.15 ^c^
Fortified soybean	α	192 ± 4.69	192 ± 7.29	182 ± 3.00	145 ± 8.79 *	158 ± 5.69 *	17.9 ± 3.00 ^b,c^
	γ	843 ± 24.0	791 ± 30.1 *	782 ± 14.9 *	655 ± 42.2 *	599 ± 18.5 *	29.0 ± 2.20 ^d^
	δ	212 ± 5.02	199 ± 0.09	193 ± 5.63 *	168 ± 10.1 *	174 ± 4.21 *	17.6 ± 2.00 ^b,c^

^e^ Data are displayed as mean ± SD (*n* = 4). Statistically significant differences were analyzed using one-way ANOVA (*p* ≤ 0.001), followed by Holm-Sidak post hoc test (*p* < 0.05). Asterisks (*) indicate significant differences within one oil type and its homolog concentration compared to day 1 (* *p* < 0.05). Different letters in the column indicate significant differences in the loss of tocopherol homologs from day 1 to day 56 in all oil types (*p* < 0.05).

**Table 4:** Correlation between the decrease in total tocopherols, the decrease in individual tocopherol homologs, the tocopherol ratio, and the increase of the POV in all four tested oils ^a^.

	Total Tocopherols	alpha-Tocopherol	gamma-Tocopherol	delta-Tocopherol	Tocopherol Ratio
	−Δ [%]	−Δ [%]	−Δ [%]	−Δ [%]	(γ + δ)/α
POV +Δ [%]	r = 0.82	r = 0.95	r = 0.08	r = −0.64	r = −0.97
	*p* < 0.001	*p* < 0.001	*p* = 0.77	*p* = 0.09	*p* < 0.001

^a^ Correlation analyses were performed at the end of the storage period (day 56) using the Pearson calculation method and the results are displayed as correlation coefficients (r) and *p* values (*p*). *p* values less than 0.05 indicate significant correlations (*n* = 4).
